# Functional Characterization of Ly49^+^CD8 T-Cells in Both Normal Condition and During Anti-Viral Response

**DOI:** 10.3389/fimmu.2020.602783

**Published:** 2021-01-07

**Authors:** Dmytro Shytikov, Deepak Rohila, Dan Li, Pengfei Wang, Mei Jiang, Mingxu Zhang, Qin Xu, Linrong Lu

**Affiliations:** ^1^ Institute of Immunology, School of Medicine, Zhejiang University, Hangzhou, China; ^2^ Zhejiang University-University of Edinburgh Institute, Zhejiang University, Haining, China; ^3^ Department of Immunology and Rheumatology in Sir Run Run Shaw Hospital, Zhejiang University School of Medicine, Hangzhou, China

**Keywords:** CD8 T-cells, memory phenotype, NK-cell receptors, Ly49 receptors, anti-viral response

## Abstract

The role of Ly49^+^CD8 T-cells in the immune system is not clear. Previously, several papers suggested Ly49^+^CD8 T-cells as immunosuppressors, while multiple studies also suggested their role as potent participants of the immune response. The mechanism of Ly49 expression on CD8 T-cells is also not clear. We investigated phenotype, functions, and regulation of Ly49 expression on murine CD8 T-cells in both normal state and during LCMV infection. CD8 T-cells express different Ly49 receptors compared with NK-cells. In intact mice, Ly49^+^CD8 T-cells have a phenotype similar to resting central memory CD8 T-cells and do not show impaired proliferation and cytokine production. Conventional CD8 T-cells upregulate Ly49 receptors during TCR-induced stimulation, and IL-2, as well as IL-15, affect it. At the same time, Ly49^+^CD8 T-cells change the Ly49 expression profile dramatically upon re-stimulation downregulating inhibitory and upregulating activating Ly49 receptors. We observed the expression of Ly49 receptors on the virus-specific CD8 T-cells during LCMV infection, especially marked in the early stages, and participation of Ly49^+^CD8 T-cells in the anti-viral response. Thus, CD8 T-cells acquire Ly49 receptors during the T-cell activation and show dynamic regulation of Ly49 receptors during stimulation.

## Introduction

CD8 T-cells express MHC class I-specific NK-cell receptors (NKRs) belonging to the killer immunoglobulin-like family of receptors (KIRs) in humans and lectin-like receptors of the Ly49 receptor family in mice (1, 2). The biological functions of these NKR^+^CD8 T-cells are obscure. Human KIR^+^CD8 T-cells are memory T-cells with an effector phenotype, which appear at certain chronic infections, such as viral infections (HIV, HCV, CMV), parasitic infections (*Trypanosoma cruzi*), or cancer (3–9). These cells show reduced effector properties, but they have survival benefits and play a role in the disease control in case of functional interactions of expressed KIRs with the corresponding MHC class I ligands (6–8). Murine Ly49^+^CD8 T-cells also represent memory T-cells (2, 10), but their biological functions are contradictory. Several studies report these cells to have efficient immunosuppressive capacities (11, 12), while other studies demonstrate the participation of Ly49^+^CD8 T-cells in the immune response and the ability to lyse virus-infected target cells (13–15).

Factors driving the expression of Ly49 receptors on CD8 T-cells are not defined yet. Numerous experiments describe the upregulation of Ly49 receptors on CD8 T-cells as a result of stimulation with the cognate antigen (either during the infection or during the exposure to self-antigens) (10, 16, 17), cytokines (18, 19), or because of interactions with activated lymphocytes during the immune response (presumably, activated CD4 T-cells) (10–12). However, the exact cellular or molecular mechanisms that govern this process, as well as the stability of Ly49 expression with time, are unknown.

Considering the uncertain role and possible implications of the NKR^+^CD8 T-cells in the immune homeostasis, a better understanding of their functions and ways of generation will help to develop treatments against viral infections, cancer, or autoimmune pathologies and improve our knowledge about the immune system. In the present study, we investigated functions and ways of generation of murine Ly49^+^CD8 T-cells in normal conditions and during virus infection using cell-based methods and LCMV infection model.

We observed the expression and dynamic regulation of Ly49 receptors on CD8 T-cells during the cell activation and assessed how IL-2 and IL-15 affect this process. We also demonstrate the positive effect of Ly49^+^CD8 T-cells from LCMV-immune mice on the antiviral response upon adoptive transfer. Our data describe cellular mechanisms of Ly49 induction on CD8 T-cells as well as demonstrate the role of Ly49^+^CD8 T-cells in the immune response.

## Materials and Methods

### Experimental Animals

C57BL/6 WT, CD45.1, RAG1 KO, Nur77^GFP+/−^ on the C57BL/6 background were obtained from the Model Animal Research Center of Nanjing University. Ly49 KO mice (C57BL/6 background) were a generous gift of Prof. Dong (Beijing). The mice were crossed with our C57BL/6 WT mice for two generations and then used for experiments. Unless indicated, experiments were performed using 6- to 12-wk-old C57BL/6 mice. Experiments were conducted under institutional guidelines for animal care and use.

### LCMV Virus Production, Quantification, and Infection

LCMV Armstrong 53b strain, BHK-21, and Vero cells were a generous gift of Prof. Ye (Chongqing). The virus was grown on BHK-21 cells and quantitated by plaque assay on Vero cells as described elsewhere (20). For acute infections, mice were injected with 2•10^5^ PFU of LCMV Armstrong i.p.

To determine LCMV titers in the spleen and liver, harvested organs were weighed, weight-adjusted to 1/10 with DMEM (Basal Media, Shanghai) with 2% FBS (Gibco) and 1% penicillin/streptomycin solution (Hangzhou Keyi Biotechnology Co., Ltd). Tissue samples were homogenized with metal beads at the Tissue Lyser 48 (Shanghai Jing Xin Industrial Development Co., Ltd) and clarified by centrifugation at 1,000 g for 10 min. Then the samples were frozen at −80°C and analyzed later. Organ homogenates were diluted in 10-fold increments and tested in the standard 4-day plaque assays.

### Antibodies, Reagents, and Equipment

Immunostaining was performed using antibodies against the following markers: CD45R (RA3-6B2), CD45RB (C363-16A), CD45.1 (A20), CD45.2 (104), TCR*β* (H57-597), CD3*ϵ* (145-2C11), CD4 (RM4-5 or GK1.5), CD8*α* (53-6.7), CD16/32 (2.4G2), CD25 (PC61), CD28 (37.51), CD44 (IM7), CD62L (MEL-14), CD69 (H1.2F3), CD122 (TM-*β*1), CD103 (2E7), CXCR3 (CXCR3-173), CTLA4 (UC10-4F10-11), PD1 (J43), KLRG1 (2F1), Lag3 (C9B7W), Tim3 (B8.2C12), Ly49A (JR9.318), Ly49C/I (5E6), Ly49C/I/F/H (14B11), Ly49G (4D11), Ly49F (HBF-719), Ly49D (4E5), Ly49H (3D10), IFN*γ* (XMG1.2), IL2 (JES6-5H4), TGF*β* (TW-716B4), Granzyme B (GB11), TNFα (MP6-XT22), IL10 (JES5-16E3).

Dead cells were excluded by the use of live/dead staining with either 7-AAD, Zombie Aqua™ fixable viability dye (Biolegend), or Fixable Viability Dye eFluor™ 450 (Invitrogen). Anti-CD16/32 (2.4G2, BD Bioscience) antibodies were used to block Fc receptors during sample preparation. Recombinant mouse IL-2 (eBioscience), recombinant mouse IL-15 (PeproTech), CellTrace™ Violet (CTV, Invitrogen), Brefeldin A (Biolegend), phorbol 12-myristate 13-acetate (PMA) and Ionomycin (Beyotime), Percoll solution (GE Healthcare) were used in experiments.

### Flow Cytometry

We used the standard protocols for flow cytometry analysis. Cells were stained with live/dead stain, incubated with Fc blocking antibodies, stained with antibodies (titrated prior experiments), and then either fixed in 2% paraformaldehyde. Cells used for staining of the intracellular antigens were stained according to the above-mentioned protocol, fixed in the IC fixation buffer (eBioscience) overnight and permeabilized with the help of 1× Permeabilization solution (eBioscience) according to the protocol and stained with the respective antibodies. Then the cells were washed once and fixed in 2% paraformaldehyde.

Samples were analyzed using ACEA NovoCyte (ACEA Biosciences) or BD Fortessa (BD Bioscience) flow cytometers and FloJo software (TreeStar, Inc). Cell sorting was done using BD FACSAria cell sorter (BD Bioscience). Ly49^+^CD8 T-cells were identified as CD8 lymphocytes that express Ly49 molecules detected by the available mAbs against Ly49A, Ly49C/F/I/H, Ly49G, Ly49F, Ly49C/I, or Ly49D+H according to the tested hypothesis.

To analyze the Ly49 repertoire expressed on CD8 T-cells, CD8 T-cells were stained with a combination of monoclonal antibodies against Ly49 receptors and subdivided according to the expression of Ly49F in 2 populations: Ly49F^+^ and Ly49F^−^. Then each of these cell populations was divided according to Ly49G expression and then the same for Ly49A and Ly49D+Ly49H. We identified Ly49^−^CD8 T-cells as the cells, which express neither of the analyzed Ly49 receptors and excluded them from the subsequent analysis. All of CD8 T-cells that express any of Ly49 receptors (even if the cell population is very small) were summarized together and considered as 100%. The amount of CD8 T-cells that express the particular combination of Ly49 receptors were expressed as a fraction of total Ly49^+^CD8 T-cells. **Figure S2** describes our gating strategy.

Similar calculations were used in assessing Ly49C/I expression. The cells were subdivided according to Ly49C/I into Ly49C/I^+^ and Ly49C/I^−^. Then each of these two populations was subdivided according to Ly49G expression. Ly49C/I^+^CD8 T-cells were used to study the co-expression pattern of the other Ly49 receptors studied (except for Ly49F) and Ly49C/I^−^G^+^ CD8 T-cells were used as a control for consistency of staining and further calculations.

### Cell Isolation

CD4 or CD8 T-cell enrichment was performed using magnetic enrichment kits (STEMCELL Technologies) according to the manufacturer instructions or with the help of biotinylated antibodies against the particular cell surface antigens (CD4, CD8*α*, CD45R, CD44, Biolegend). The cell suspension was adjusted to 100•10^6^/ml, incubated with rat serum (STEMCELL Technologies), antibodies at the desired concentration (titrated before experiments), washed three times, and incubated with MojoSort streptavidin nanobeads (Biolegend) followed by incubation on a magnet (STEMCELL Technologies). Enrichment efficacy of the desired cell fraction was assessed using flow cytometry. For fluorescence-associated cell sorting, cells were incubated with fluorochrome-labeled antibodies 20 min on ice as described and sorted (FACSAria, BD Bioscience).

Intrahepatic lymphocytes were isolated according to the commonly accepted method (21). The resulting cell suspensions were mixed with 40% isotonic Percoll and centrifuged on the gradient of 70% isotonic Percoll at 600 g RT for 30 min without break. Mononuclear cells were collected from the interphase between both layers. The cells were used for flow cytometry analysis (TCR*β* ^+^CD8*α* ^+^ cells).

Peripheral blood lymphocytes were collected from the saphenous vein into the tubes with EDTA at the final concentration of 5 mM. The resulting suspension was additionally diluted with PBS and centrifuged on 70% isotonic Percoll for 30 min at 600 g RT without break. Mononuclear cells were collected from the interphase between the layers.

### T-Cell Proliferation *In Vitro*

Naïve CD8 T-cells (CD8^+^CD44^low^Ly49^−^, nCD8), conventional memory phenotype CD8 T-cells (CD8^+^CD44^high^Ly49^−^, MP CD8), and Ly49^+^CD8 cells were isolated by FACS and stained with CellTrace™ Violet or left unstained. The cells were stimulated with plate-bound anti-CD3ϵ (0.03 to 3 mcg/ml coated overnight) and soluble anti-CD28 antibodies (0.4 mcg/ml) alone or in combinations with IL-2 (25–100 U/ml) or with IL-15 (50–100 ng/ml) or just with the cytokines for 48–72 h. Proliferation and changes in the surface phenotype were assessed.

Cells were cultured in 96-well round-bottom plates at 37°C in 5% CO_2_ in RPMI-1640 (Basal Media, Shanghai) supplemented with 10% FBS (Gibco), 10 mM HEPES (Solarbio), 1 mM sodium pyruvate (Sigma-Aldrich), 50 nM beta-mercaptoethanol (Solarbio), 100 units/ml of penicillin, 0.1 mg/ml streptomycin (Hangzhou Keyi Biotechnology Co., Ltd).

### Intracellular Cytokine Staining

Cytokine production by FACS-purified CD8 T-cells from naïve mice was measured using polyclonal stimulation. Sorted cell subsets were stimulated with plate-bound anti-CD3ϵ and soluble anti-CD28 (1 mcg/ml coated overnight and 0.4 mcg/ml, respectively) or with IL-15 (50 ng/ml) for 3 days. Then the cells were re-stimulated with PMA and ionomycin (final concentration is 50 ng/ml and 500 ng/ml, respectively) or left untreated (only applicable for the cells cultured on IL-15, which were used as a control), following with the addition of Brefeldin A for 4 h.

LCMV-specific memory CD8 T-cells were detected by measuring intracellular cytokine production in response to immunodominant LCMV peptides (gp33, KAVYNFATCGI; gp276, SGVENPGGYCL; np205, YTVKYPNI; np396, FQPQNGQFI; Sangon). Briefly, 3–7•10^6^ splenocytes were incubated in 96-well plates (1 h, 37°C) with 5 mcM synthetic peptides. Then Brefeldin A was added and the cells were incubated for another 4 h at 37°C with 5% CO_2_.

### Adoptive Transfer Experiments

#### Upregulation of Ly49 Markers on CD8 T-Cells During Homeostatic Expansion

Upregulation of Ly49 markers on CD8 T-cells during homeostatic expansion was measured by the adoptive transfer of 0.2•10^6^ sorted CD8 T-cells alone or together with 0.5•10^6^ sorted CD4 T-cells. FACS-purified cell subsets were injected *i.v.* into RAG1 KO mice. Blood collection was made monthly for 5 months from the saphenous vein. Erythrocyte-depleted PBLs were stained with fluorochrome-labeled antibodies against TCR*β*, CD4, CD8, CD44, and Ly49. The phenotype of T-cells was analyzed. 5 months later the mice were euthanized, splenocytes were collected, and the phenotype of T-cells was assessed.

#### Anti-Viral Protection

Protective functions of different subsets of CD8 T-cells were studied using the adoptive transfer of IL-15-expanded memory CD8 T-cells from LCMV-immune mice into naïve hosts. Cell subsets from LCMV-immune mice (2 months after infection) were FACS-purified and expanded on IL-15 (100 ng/ml) for 2 weeks. The cells were checked to confirm phenotype and viability and 2•10^6^ cells of each type were injected i.v. into naïve WT hosts. Control mice received i.v. injection with HBSS. The same day those mice were infected with LCMV Armstrong and the mice were sacrificed 5 days later.

#### Upregulation of Ly49 Markers on CD8 T-Cells During the Antiviral Response

Upregulation of Ly49 markers on CD8 T-cells during the antiviral immune response was assessed by the adoptive transfer of naïve WT CD45.2 naïve CD8 T-cells alone or together with total CD45.1 CD8-depleted splenocytes into sublethally irradiated (2.5 Gy, RS-2000, Rad Source Technologies) congenic CD45.1 mice. The recipients were either left untreated or infected with LCMV Armstrong.

Naïve WT CD45.2 CD8 T-cells were obtained by magnetic enrichment using biotinylated antibodies against CD4, CD44, and CD45R as described above. Cells were >90% CD8, >99% Ly49^–^, >97% CD44^low^ as measured by flow cytometry. Total CD45.1 CD8-depleted splenocytes were prepared similarly, but the donor cells were incubated with biotinylated antibodies against CD8α. The resultant cells contained <1% CD8 cells as measured by flow cytometry.

Irradiated recipient mice received 5•10^6^ WT CD45.2 naïve CD8 T-cells alone or together with total CD45.1 CD8-depleted splenocytes (at least 5•10^6^ CD4 cells and 30•10^6^ B-cells per recipient). Mice received 2•10^5^ PFU of LCMV Armstrong or PBS i.p. the next day after cell transfer. Cell phenotype of transferred CD8^+^ T-cells and their antigen-specific cytokine production was assessed as described above.

### Statistical Analysis

Statistical analysis was conducted using Statistica (Statsoft), GraphPad (Prism) or MS Excel (Microsoft). The equality of variances between experimental groups was tested using F-test or Levene’s test prior to further analysis. Each data set was subjected to two-sided Student *t*-test or ANOVA with subsequent Newman–Keuls *post-hoc* tests. We used the last observation carried forward imputations method to deal with dropouts measuring mouse body weight changes during the colitis prevention study. The data is presented as the mean ± the standard error of the mean. *P- value <*0.05 was considered significant.

## Results

### Ly49^+^CD8 T-Cells Have the Phenotype of Resting Central Memory CD8 T-Cells and Express Ly49 Receptors in a Combinatorial Way

**Figure 1:**
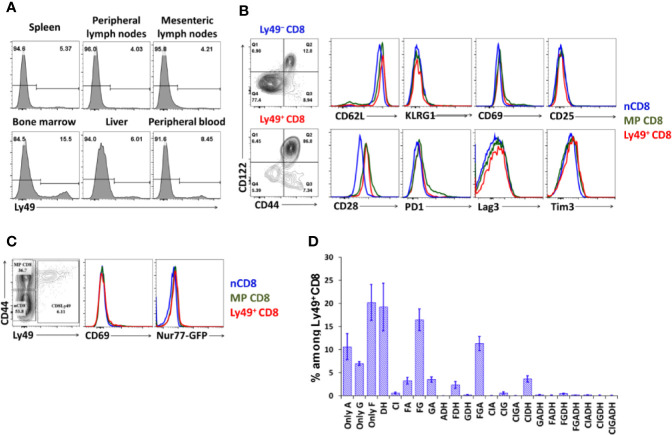
Organ distribution and cell phenotype of Ly49^+^CD8 T-cells. **(A)** Lymphocytes from the respective organs were stained with antibodies against CD8, TCR*β*, and inhibitory Ly49 receptors. Numbers represent the percentage of Ly49^+^CD8 T-cells among the total CD8 cells. Plots are representative of five mice. **(B)** Splenocytes were stained with antibodies against CD8, CD44, CD122, inhibitory Ly49 receptors and the indicated surface markers. Plots are representative of five mice. **(C)** CD69 and Nur77 expression on naïve, memory phenotype and Ly49^+^CD8 T-cells from naïve Nur77^GFP+/−^. Plots are representative of three mice. **(D)** The repertoire of Ly49 receptors on splenic Ly49^+^CD8 T-cells. The data is summarized of four mice.

We isolated and checked distribution and cell surface phenotype of Ly49^+^CD8 T-cells from spleen, pooled peripheral lymph nodes, mesenteric lymph nodes, bone marrow, peripheral blood, and liver. Depending on the site analyzed (**Figure 1A**), the amount of Ly49^+^CD8 T-cells among total CD8 T-cells varies from 4 to 8% in lymph nodes and spleen with only a slightly higher percentage in the liver and significant enrichment within the bone marrow compared with the other organs analyzed (**Figure 1A**). Ly49^+^CD8 T-cells show quite a uniform cell surface phenotype among the organs tested, so we concentrated our attention mainly on the splenic population of Ly49^+^CD8 T-cells. The cell surface phenotype of Ly49^+^CD8 T-cell suggests that these cells are very close to conventional memory phenotype CD8 T-cells, not showing exhaustion or excessive cell activation (**Figure 1B**). Additionally, Ly49^+^CD8 T-cells do not express Nur77 expression in naïve Nur77^GFP+/−^ mice (**Figure 1C**).

CD8 T-cells express a wide variety of combinations of Ly49 receptors (**Figure 1D**, gating strategy is described in the relevant section of *Materials and Methods* and depicted in **Figure S1**) with the absolute predominance of Ly49F^+^ cells. The next most expressed receptors are Ly49G and Ly49A, which are mostly expressed together with Ly49F (16.4% of Ly49^+^CD8 T-cells are Ly49F^+^G^+^, 3.2% are Ly49F^+^A^+^, and ~11.3% are Ly49F^+^G^+^A^+^). Ly49C and Ly49I are the least expressed inhibitory receptors among tested. Cumulatively, 26% of total Ly49^+^CD8 T-cell express activating Ly49D and Ly49H receptors mostly without other inhibitory Ly49 receptors. Only a minority of Ly49^+^CD8 T-cell co-express inhibitory and activating receptors in naïve mice.

### CD8 T-Cells Acquire Ly49 Receptors Post-Thymically

**Figure 2:**
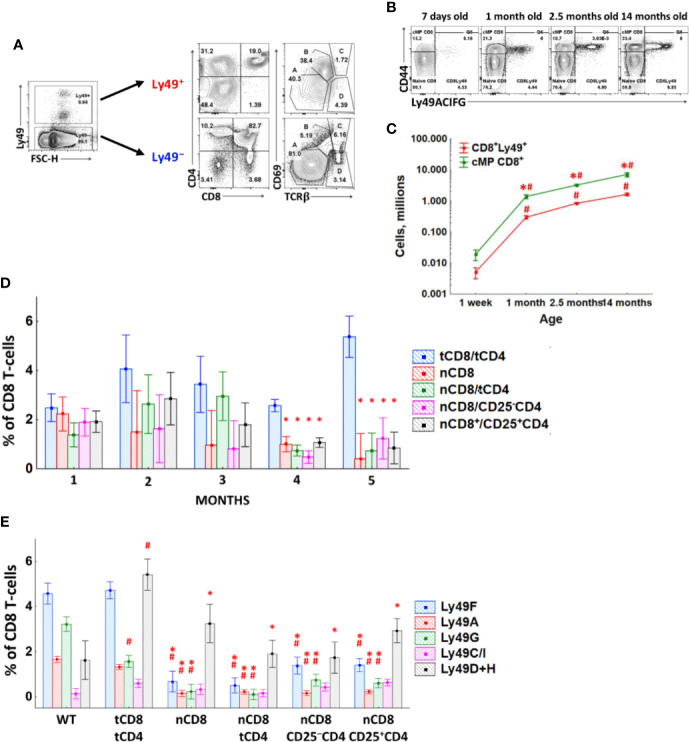
Generation of Ly49^+^CD8 T-cells. **(A)** Thymocytes were divided according to Ly49 expression and stained with antibodies against CD4, CD8*α*, CD69, and TCR*β*. Plots are representative of four mice. **(B)** Splenic CD8 T-cells of mice of different ages were analyzed for the amount of Ly49^+^CD8 T-cells. Representative plots of mice from each age group are shown. **(C)** The absolute quantity of Ly49^+^ and Ly49^−^ splenic memory phenotype CD8 T-cells of mice of different ages. Plots are representative of three separate experiments with at least three mice per age group. *P_(t)_ < 0.05 comparing to another cell type at the particular time point. ^#^P_(t)_ < 0.05 comparing to the previous time-point. **(D)** The amount of Ly49^+^CD8 T-cells among total peripheral blood CD8 T-cells of RAG1 KO mice injected with different types of CD8 or CD4 T-cells. **(E)** The amount of CD8 T-cells expressing the particular Ly49 receptor among total splenic CD8 T-cells of RAG1 KO mice injected with different types of CD8 or CD4 T-cells, 5 months after the injection. Each group includes at least three mice. *P_(t)_ < 0.05 comparing to the group tCD8/tCD4. ^#^P_(t)_ < 0.05 comparing to intact WT mice.

We subdivided thymocytes according to the expression of inhibitory Ly49 receptors into Ly49^−^ and Ly49^+^ and checked for CD4, CD8*α*, CD69, and TCR*β* receptors. Ly49^+^ thymocytes, on average, account for less than 0.5% of the cells, while in the periphery, 4–7% of mature CD8 T-cells express Ly49 receptors. Most of the Ly49^+^ thymocytes are CD4^+^CD8^+^, CD4^−^CD8^−^, and CD4^+^CD8^−^ thymocytes, which may represent developing NK T-cells (**Figure 2A**), while there is no distinct Ly49^+^CD4^−^CD8^+^ population.

One-week-old mice show a percentage of Ly49^+^CD8 T-cell comparable with adult mice, and this percentage is relatively stable throughout life (**Figure 2B**). Only the advanced age mice accumulate Ly49^+^CD8 T-cells significantly, but it is accompanied by a much more pronounced expansion of Ly49^−^ memory phenotype CD8 T-cells. The absolute number of Ly49^+^CD8 T-cells grows with age gradually, but there is no selective accumulation of exactly this cell population (**Figure 2C**).

To assess the involvement of autoantigens and/or cytokines on Ly49 induction, we injected lymphopenic RAG1 KO mice with naïve CD8 T-cells (nCD8) alone or together with different populations of CD4 T-cells (total peripheral CD4 T-cells (tCD4), or CD25^+^CD4 T-cells, or CD25^−^CD4 T-cells). RAG1 KO mice injected with total CD8 and total CD4 T-cells (tCD8/tCD4) were used as a control group. Injected T-cells repopulated RAG1 KO mice successfully and acquired CD44 (data not shown). But naïve CD8 T-cells from all of the experimental group upregulated Ly49 receptors only on a very small number of cells (**Figure 2D**), while pre-existing Ly49^+^CD8 T-cells (RAG1 KO mice injected with total CD8 and total CD4 T-cells) preserved Ly49 expression on their surface and mostly retained their quantity stably. Only a fraction of CD8 T-cells acquired Ly49 receptors by the end of the study period, and these were activating Ly49 receptors (**Figure 2E**).

### Ly49+CD8 T-Cell Do Not Have Proliferation Defects

**Figure 3:**
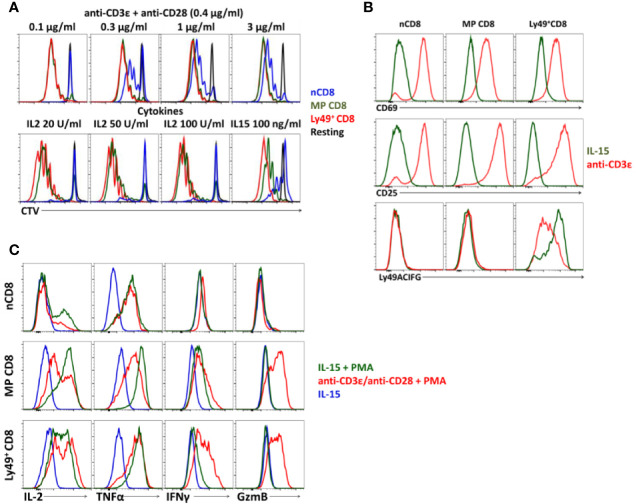
Cell phenotype of Ly49^+^CD8 T-cells. **(A)** Proliferation of FACS-purified naïve (nCD8), memory phenotype (MP CD8) and Ly49^+^CD8 T-cells. Plots are representative of five independent experiments. **(B)** Expression of the activation markers and inhibitory Ly49 receptors on CD8 T-cells during TCR stimulation or culture on IL-15. Plots are representative of four independent experiments. **(C)** Expression of cytokines and Granzyme B by different populations of CD8 T-cells upon stimulation at the indicated conditions *in vitro*. Plots are representative of three independent experiments.

Ly49^+^CD8^+^ T-cells demonstrate proliferation comparable to conventional memory phenotype CD8 T-cells (**Figure 3A**) and proper upregulation of CD25 and CD69 in response to anti-CD3*ϵ*/anti-CD28 antibodies or IL-15 *in vitro* (**Figure 3B**). At the same time, we noticed a considerable reduction in expression of inhibitory Ly49 receptors (cells were stained with a mixture of antibodies against Ly49C/F/I/H, Ly49A, Ly49G) on the surface of Ly49^+^CD8 T-cells upon TCR stimulation while stimulation with IL-15 did not have such an effect (**Figure 3B**).

Upon activation, Ly49^+^CD8 T-cells express pro-inflammatory cytokines (IL2, TNFα, and IFN*γ*) and granzyme B at the level comparable to the conventional memory CD8 T-cells (**Figure 3C**) and do not express IL10 or TGF*β* (data not shown).

### Ly49+CD8 T-Cells Participate in the Antiviral Respons

**Figure 4:**
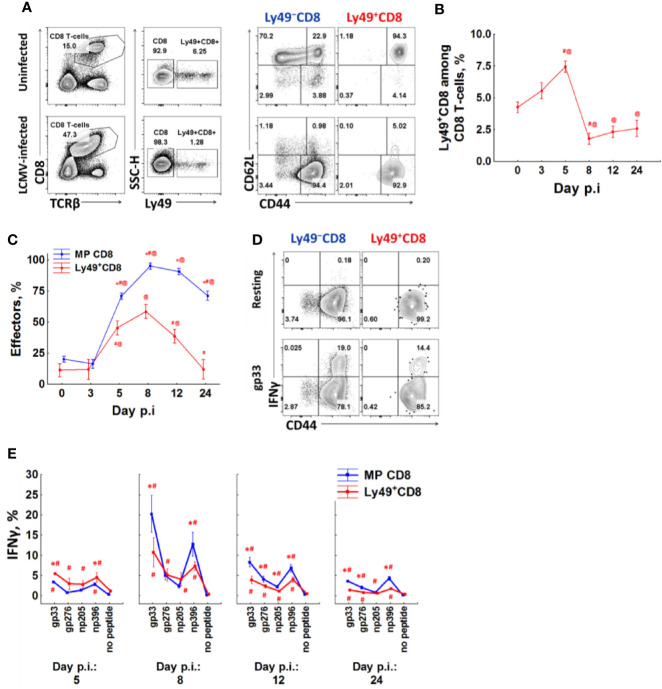
A fraction of anti-viral effector CD8 T-cells upregulates Ly49 receptors. **(A)** Surface phenotype of splenic CD8 T-cells from intact or LCMV-infected (day 8) mice. Representative pictures of each group are shown. **(B)** The percentage of CD8 T-cells expressing inhibitory Ly49 receptors among the total splenic CD8 T-cells at different time points after infection. **(C)** the relative amount of effector cells (CD44^high^CD62L^low^) among Ly49^+^ and CD44^high^Ly49^−^ of CD8 T-cells at different time points after infection. The data is summarized from two independent experiments, each time point of each experiment has at least four mice. *P_(t)_ < 0.05 comparing to Ly49^+^CD8 T-cells. ^#^P_(t)_ < 0.05 comparing to the same type of CD8 T-cells at the previous time point. ^@^P_(t)_ < 0.05 comparing to the same type of CD8 T-cells from non-infected mice. **(D)** IFN*γ* expression by different CD8 T-cell populations during peptide re-stimulation *in vitro* (day 8 post-infection). Representative plots of each cell population are shown. **(E)** The relative amount of IFN*γ* ^+^ cells among each type of CD8 T-cells. Each time point includes at least four mice. ^*^P_(t)_ < 0.05 comparing to another type of CD8 T-cells. ^#^P_(t)_ < 0.05 comparing to the same type of CD8 T-cells which were left resting (no peptide).

We infected mice with LCMV Armstrong and checked T-cell phenotype and IFN*γ* expression at different time points (3, 5, 8, 12, and 24 days after infection). 5 days post-infection, CD8 T-cells demonstrate pronounced activation and expansion of memory CD8 T-cells and their differentiation towards the effector phenotype (**Figures 4A, C**, and **S2A**). The relative amount of Ly49^+^CD8 T-cells also increases, but modestly (from 4.25% to more than 7.5% among all CD8 T-cells, P_(t)_ <0.05, **Figures 4A, B**, and **S2A**), and they also show effector differentiation (**Figure 4C**). At this time point, the relative amount of IFN*γ* producers among Ly49^+^CD8 T-cells is higher than among conventional memory phenotype CD8 T-cells (P_(t)_ <0.05, **Figures 4D, E**, and **S2B**). Overall, Ly49^+^CD8 T-cells account for approximately 10% of total IFN*γ* producers in response to viral peptides (data not shown) at this time point. 8 days post-infection effector CD8^+^ T-cells expand greatly (**Figure 4C**), and Ly49^+^CD8 T-cells are outnumbered by the conventional memory CD8^+^ T-cells (**Figure 4C** and **S2A**). Ly49^+^CD8 T-cells also contain a high proportion of IFN*γ* producers (**Figures 4D, E**, and **S2B**).

Ly49^+^CD8 T-cells contract and reacquire CD62L expression faster than conventional memory CD8 T-cells (**Figures 4B, C**). The percentage of Ly49^+^CD8 T-cells approaches the one of non-infected mice only 24 days after infection (**Figure 4B**). The amount of IFN*γ* producers also decreases, but they are still detectable even 24 days after infection (1.43% of Ly49^+^CD8 T-cells produce IFN*γ* in response to gp33 comparing to 3.6% of conventional memory CD8 T-cells; **Figures 4E** and **S2B**).

To check whether the absence of Ly49^+^CD8 T-cells as well as the possibility to acquire the Ly49 receptors, is beneficial for CD8 T-cells or not, we assessed the phenotype of mice completely lacking the whole cluster of Ly49 genes.

**Figure 5:**
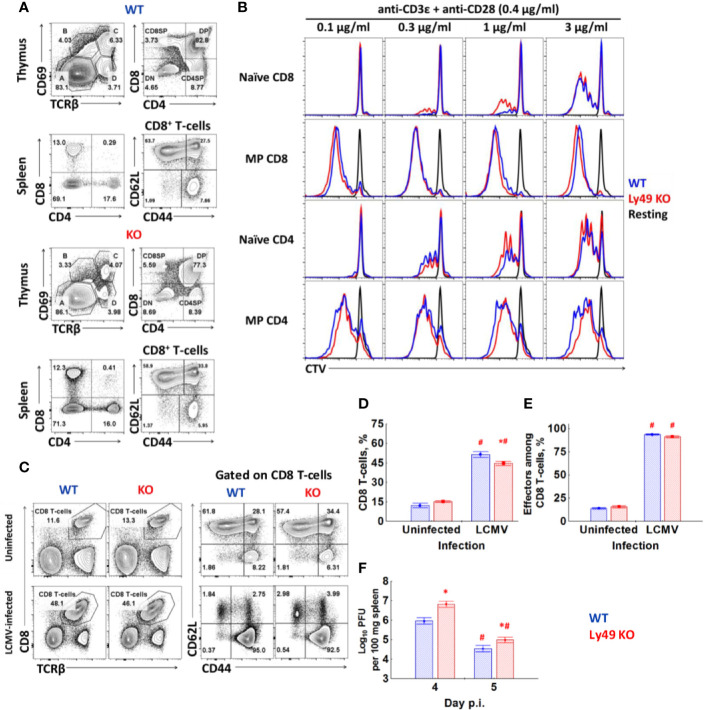
Ly49 KO mice don’t show major defects in T-cell development or T-cell response to virus infection. **(A)** Surface phenotype of splenocytes and thymocytes of WT or Ly49 KO mice stained with antibodies against CD8, CD4, TCR*β*, CD69, CD44. Plots are representative from four mice. **(B)** FACS-purified naïve and memory phenotype T-cells from each type of mice were stained with CTV and stimulated *in vitro* with the indicated concentrations of antibodies. Plots are representative of five independent experiments. **(C)** Surface phenotype of splenocytes from intact or LCMV-infected (day 8) WT and Ly49 KO mice. **(D)** The percentage of CD8 T-cells among the total splenocytes of intact or LCMV-infected WT or Ly49 KO mice. **(E)** The relative amount of effector cells (CD44^high^CD62L^low^) among CD8 T-cells of mice of each type. ^*^P_(t)_ < 0.05 comparing to WT mice. ^#^P_(t)_ < 0.05 comparing to intact mice of the same genotype. **(F)** Viral titer in the spleen of WT or Ly49 KO mice on days 4 and 5 after infection. *P_(t)_ < 0.05 comparing to WT mice. ^#^P_(t)_ < 0.05 comparing to the same type of mice at the previous time point. The data is summarized from two experiments; each experimental group includes at least four mice.

Ly49 KO mice do not show significant differences in the thymopoiesis or ratios of major peripheral T-cell subsets compared to WT (**Figure 5A**). We found only marginal differences in the proliferation of naïve CD8 T-cells during the polyclonal stimulation *in vitro* (**Figure 5B**). 8 days post-infection with LCMV Ly49 KO demonstrate the marginally less pronounced expansion of CD8 T-cells (**Figures 5C–E**), acquisition of KLRG1 by CD8 T-cells (**Figure S3A**), and slightly higher viral titer in the spleen on days 4 and 5 (**Figure 5F**). Overall, cell dynamics and cytokine production are not perturbed in CD8 T-cells from Ly49 KO mice (**Figures S3B–D**).

We assessed the role of Ly49^+^CD8 T-cells in the secondary immune response using the adoptive transfer of memory CD8 T-cells from LCMV-immune mice (Ly49^–^CD44^+^CD8 or Ly49^+^CD8 cells from WT mice or total CD44^+^CD8 cells from Ly49 KO mice) into naïve WT hosts, which were infected subsequently. As we intended to preserve the antigen specificity of Ly49^+^CD8 T-cells avoiding the selection of particular clones, we expanded those populations of CD8 T-cell using IL-15. According to our (**Figure 3A**) and previously published observations Ly49^+^CD8 T-cells expand nicely in response to this cytokine (12).

**Figure 6:**
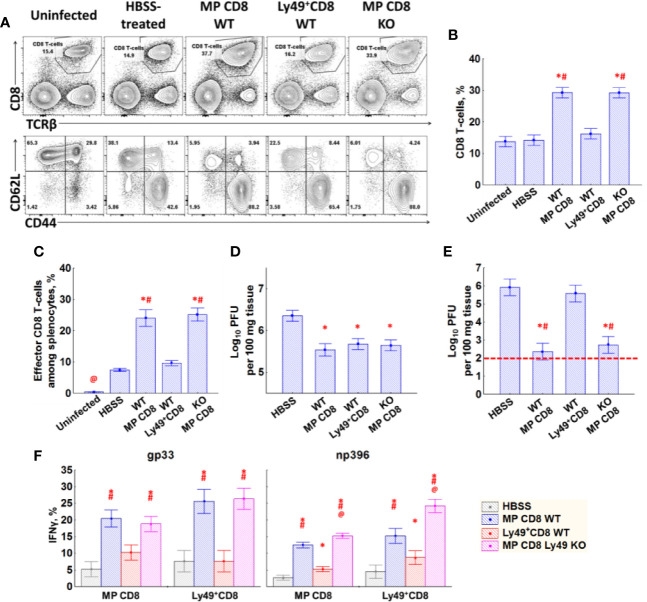
Effects of transfer of Ly49^−^ memory CD8 T-cells, Ly49^+^CD8 T-cells from LCMV-immune WT mice or total memory CD8 T-cells from LCMV-immune Ly49 KO mice. **(A)** Splenocytes of mice (day 5 after injection) of each type were stained with antibodies against TCR*β*, CD8*α*, CD44 and CD62L. Representative pictures of mice from each group are shown. **(B)** The relative amount of CD8 T-cells among the total splenocytes. **(C)** The relative amount of effector memory (CD44^high^CD62L^low^) CD8 T-cells among the total splenocytes. **(D)** Viral titer in the spleen. **(E)** Viral titer in the liver. Red dotted line represents the detection limit. **(F)** IFN*γ* expression by memory phenotype CD8 T-cells and Ly49^+^CD8 T-cells in response to gp33 (left graph) and np396 (right graph). Each experimental group includes five mice. *P_(t)_ < 0.05 comparing to HBSS-treated LCMV-infected mice. ^#^P_(t)_ < 0.05 comparing to LCMV-infected mice that received Ly49^+^CD8 T-cells from WT LCMV-immune mice. ^@^P_(t)_ < 0.05 comparing to the other experimental groups.

Mice injected with WT Ly49^−^ memory phenotype CD8 T-cells and total memory phenotype CD8 T-cells from Ly49 KO mice showed robust expansion of effector CD8 T-cells (**Figures 6A–C**), decreased viral titer in the spleen and liver (**Figures 6D, E**) and an increased amount of IFN*γ* producing cells in the spleen (**Figure 6F**). Ly49^+^CD8 T-cells from WT LCMV-immune mice did not affect the antiviral response that much strong, yet still evident. The relative increase in effector CD8 T-cells was small but evident (54.6% of effector cells among memory phenotype CD8 T-cells in HBSS-treated mice *vs* 61.2% in Ly49^+^CD8-treated animals, P_(t)_<0.05; data not shown). Ly49^+^CD8 T-cells of the mice injected with Ly49^+^CD8 T-cells from WT LCMV-immune mice showed more active effector differentiation compared with the HBSS-treated mice (58.8% of effector cells among Ly49^+^CD8 T-cells in HBSS-treated mice *vs* 70.8% in Ly49^+^CD8-treated animals, P_(t)_<0.05; data not shown). Moreover, Ly49^+^CD8 T-cells showed protective properties as far as their transfer reduced viral load in the spleens of infected animals compared with the control group (**Figure 6D**) and increased the amount of IFN*γ* producing cells among CD8 T-cells (**Figure 6F**).

### Activation-Induced Alterations of Ly49 Expression on CD8 T-Cells

Sorted populations of CD8 T-cells from naïve WT mice were stimulated with anti-CD3*ϵ* and anti-CD28 antibodies alone or in the presence of IL-15 or IL-2 or with cytokines only for 3 days *in vitro* to assess changes in Ly49 expression profile.

**Figure 7:**
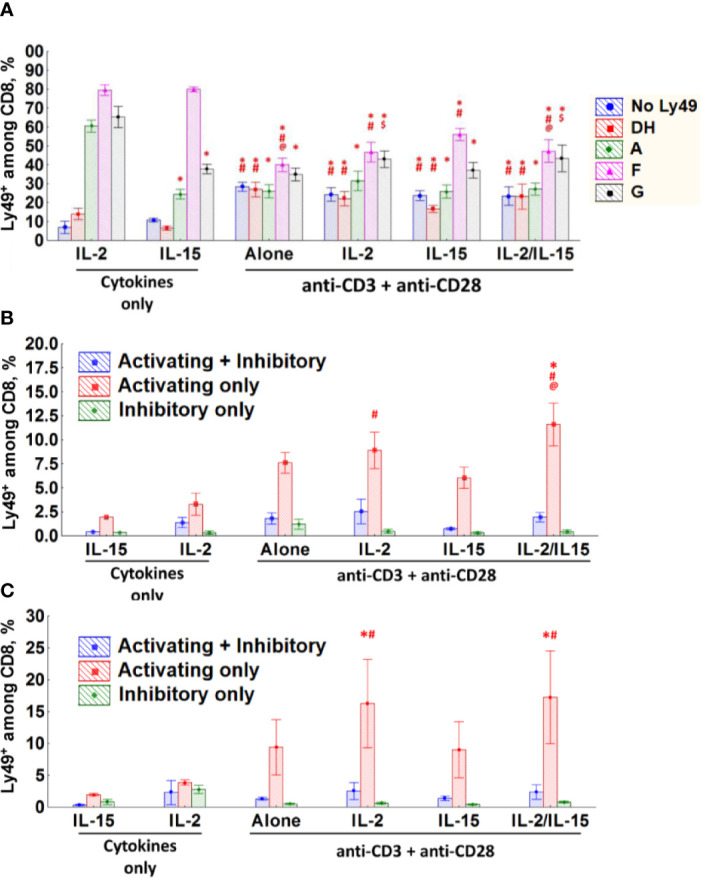
CD8 T-cells express different Ly49 receptors upon stimulation *in vitro*. **(A–C)** The percentage of Ly49^+^CD8 T-cells **(A)**, naïve **(B)** or memory phenotype **(C)** CD8 T-cells expressing the respective combinations of Ly49 receptor after stimulation during 72 h at the indicated experimental conditions *in vitro*. Data collected from six separate experiments. *P_(t)_ < 0.05 comparing to the IL-2-cultured cells. ^#^P_(t)_ < 0.05 comparing to the IL-15-cultured cells. ^@^P_(t)_ < 0.05 comparing to the cells stimulated with anti-CD3ϵ, anti-CD28 and IL-15. ^$^P_(t)_ < 0.05 comparing to the cells stimulated with anti-CD3ϵ and anti-CD28.

TCR stimulation of Ly49^+^CD8 T-cells reduced the expression of inhibitory Ly49 receptors, especially Ly49F, significantly while upregulating activating receptors (**Figure 7A**, **Figure S4A**, and **Table S1**). IL-15 supports the expression of Ly49F alone or together with other inhibitory Ly49 receptors (Ly49F^+^A^+^, Ly49F^+^G^+^, Ly49F^+^G^+^A^+^) while keeping the expression of activating Ly49 receptors low. Ly49^+^CD8 T-cells preserve the expression of Ly49F and show reduced expression of activating receptors if they are TCR stimulated in the presence of IL-15. Strikingly, Ly49^+^CD8 T-cells cultured on IL-2 show the considerable accumulation of Ly49G^+^ cells, usually in combination with Ly49F and Ly49A (**Figure 7A**, **Figure S4A**, and **Table S1**). Simultaneous TCR and cytokine stimulation of Ly49^+^CD8 T-cells led to the combined effect of both stimuli.

As conventional CD8 T-cells do not upregulate inhibitory Ly49 receptors massively (**Figure 3B**) we grouped the cells according to the expression of any inhibitory (Ly49A, G, F, and C/I) or activating (Ly49D and H) receptors or both types together. While the conventional CD8 T-cells (naïve and Ly49^–^ memory phenotype) express low levels of inhibitory Ly49 receptors upon TCR stimulation, there is an evident increase in expression of activating Ly49 receptors (**Figures 7B, C**, **S4B, C**). Major factors affecting the expression of activating Ly49 receptors on naïve CD8 T-cells included stimulation strength (data not shown) as well as the presence of IL-2 and IL-15 in the culture medium (**Figure 7B**). Memory phenotype CD8 T-cells also upregulate activating Ly49 receptors upon TCR ligation, but we did not find strong dependence on the strength of TCR stimulation or the presence of cytokines (**Figure 7C**).

**Figure 8:**
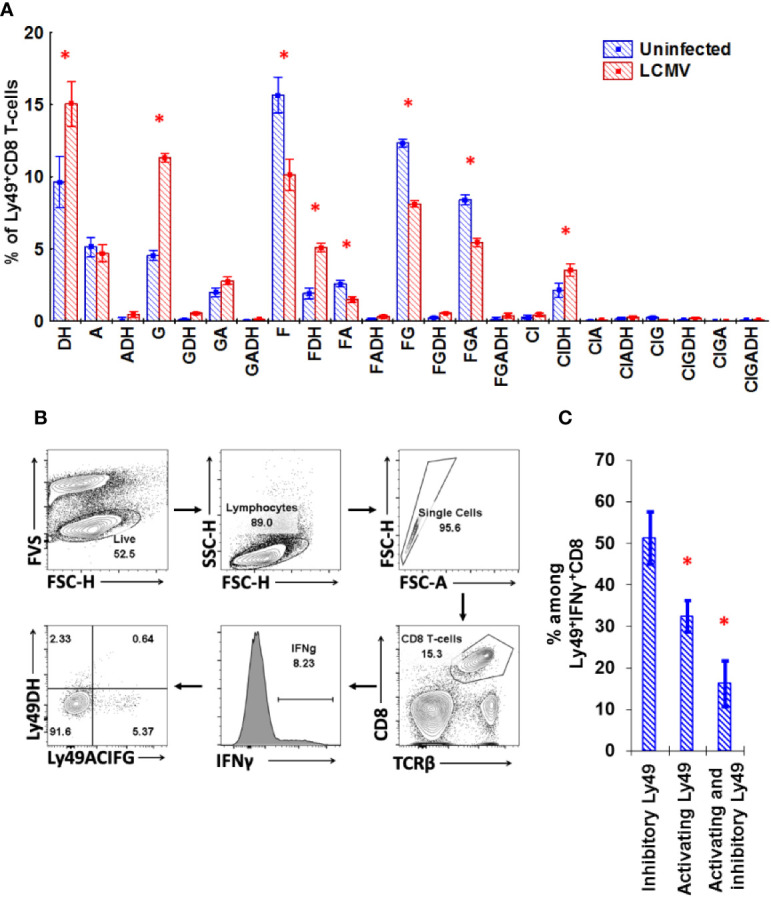
Ly49 repertoire of CD8 T-cell changes during LCMV infection significantly. **(A)** The relative amount of splenic Ly49^+^CD8 T-cells expressing the particular combinations of Ly49 receptors from naïve or LCMV-infected mice. *P_(t)_ < 0.05 comparing to naïve mice. The data is summarized from two independent experiments, and each experimental group includes four mice. **(B)** Gating strategy used to determine the repertoire of Ly49 receptors expressed on the IFN*γ*-producing CD8 T-cells during the primary LCMV infection. CD8 T-cells that showed detectable production of IFN*γ* in response to the combination of gp33 and np396 at 5 mcg/ml were selected and these IFN*γ* ^+^ cells were divided according to the expression of Ly49A+C/I+F+G and Ly49D+H. Frequency of each population was determined and calculated. The representative plot is presented. **(C)** The relative amount of CD8 T-cells expressing the particular Ly49 receptors among CD8 T-cells producing IFN*γ* in response to the mixture of gp33 and np396 peptides. *P_(t)_ < 0.05 comparing to the cell that express inhibitory Ly49 receptors. The sample includes four mice.

To confirm our results with *in vitro* stimulation, we tested the impact of LCMV infection on the repertoire of Ly49 receptors expressed on CD8 T-cells. During LCMV infection, the Ly49 repertoire of CD8 T-cells changes significantly. We observed an increase in the percentage of Ly49D/H^+^ and Ly49G^+^ CD8 T-cells and a decrease for the other combinations of Ly49 receptors, especially those including Ly49F (Ly49F^+^, Ly49F^+^A^+^, Ly49F^+^G^+^, Ly49F^+^G^+^A^+^; **Figure 8A**). IFN*γ* producers among Ly49^+^CD8 T-cells express both inhibitory and activating Ly49 receptors, but those cells expressing inhibitory receptors account for a significantly higher percentage (**Figure 8C**; gating strategy is depicted in **Figure 8B**).

**Figure 9:**
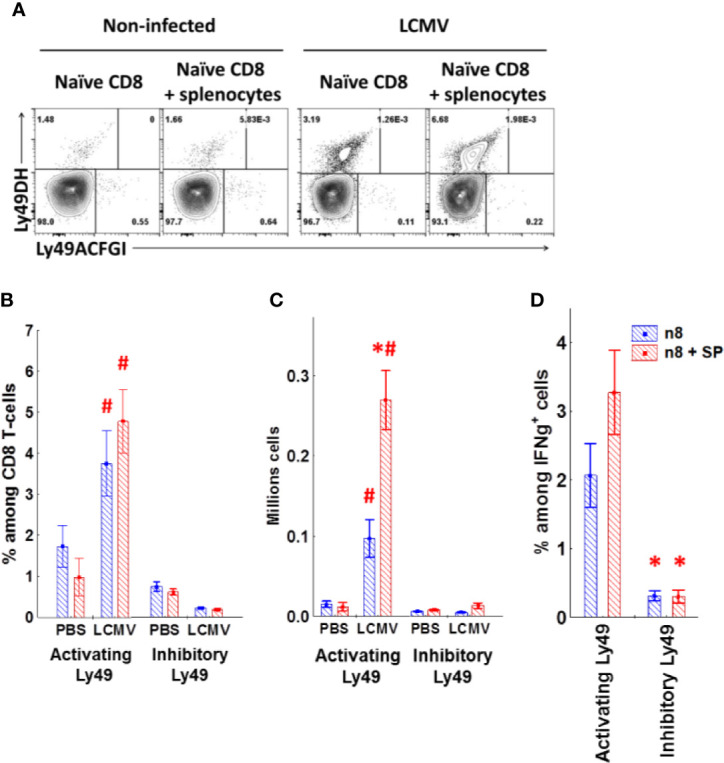
Naïve CD8 T-cells upregulate activating Ly49 receptors during virus infection. **(A)** The expression of activating (DH) or inhibitory (ACFGI) Ly49 receptors on CD45.2 naïve CD8 T-cells after the adoptive transfer alone or with CD45.1 splenocytes depleted of CD8 T-cells into sublethally irradiated (2.5 Gy) CD45.1 mice. The recipient mice were either left non-treated or infected with LCMV. Representative plots are shown. **(B**, **C)** The relative amount and absolute cell number of transferred CD45.2 CD8 T-cells expressing different types of Ly49 receptors under the indicated experimental conditions. ^*^P_(t)_ <0.05 comparing to the mice which received only naïve CD8 T-cells. ^#^P_(t)_ < 0.05 comparing to non-infected mice. **(D)** the relative amount of the transferred CD45.2 CD8 T-cells secreting IFN*γ* and expressing the particular types of Ly49 receptors among the total CD8 T-cells producing IFN*γ* in response to peptide stimulation. *P_(t)_ < 0.05 comparing to the cells expressing activating Ly49 receptors. The data is summarized from three independent experiments, and each group includes at least three mice in each experiment.

To confirm the antigen-induced upregulation of Ly49 receptors, we transferred naive CD8 T-cells alone or with the total splenocytes depleted of CD8 T-cells into sublethally irradiated congenic mice. Only a few of the transferred cells upregulated activating Ly49 receptors in non-infected mice (**Figures 9A–C**) with no induction of inhibitory Ly49 receptors regardless of the presence of CD8-depleted splenocytes. Strikingly, transferred CD8 T-cells acquired activating Ly49 receptors under the infection conditions (**Figures 9A, B**) and expanded actively (**Figure 9C**). The presence of CD8-depleted splenocytes facilitated the expansion of Ly49DH^+^CD8 T-cells as well as the amount of the antigen-specific cells able to produce IFN*γ* in response to the viral peptides (**Figure 9D**) while not affecting the quantity and percentage of Ly49ACIFG^+^CD8 T-cells.

## Discussion

Our data suggest that murine CD8 T-cells upregulate Ly49 receptors upon activation. These Ly49^+^CD8 T-cells lack immunosuppressive activity and, instead, arise during the antiviral response and contribute to it, although, are not critical participants. We observed stepwise acquisition of Ly49 receptors by CD8 T-cells. Overall, Ly49 expression on CD8 T-cells is not stable, but rather a dynamic process, which depends on TCR stimulation and factors secreted by the microenvironment. Common *γ*-chain cytokines, IL-2 and IL-15, affect Ly49 expression.

Organ distribution and cell surface phenotype of Ly49^+^CD8 T-cells are similar to the conventional central memory CD8 T-cells, showing high expression of CD122, CD44, CD62L, and absence of markers showing activation (CD69, CD25, KLRG1, Nur77) or inhibitory receptors (Lag3, PD1, Tim3). Ly49^+^CD8 T-cells mostly express Ly49F, Ly49A, and Ly49G along with a handful of expression of activating receptors, Ly49D and Ly49H. This expression profile contrasts with the Ly49 repertoire of NK-cells described before, which highly express Ly49D+H alone or together with Ly49G or Ly49A and minutely express Ly49F (22, 23). It is interesting as according to these data the majority of Ly49 receptors expressed on Ly49^+^CD8 T-cells do not have strong MHC class I ligands among the MHC class I proteins expressed in H2^b^ hosts. It is a quite notable similarity between murine Ly49^+^CD8 and human KIR^+^CD8 T-cells: receptors expressed by both cell types usually do not match with the host NK-cells (24, 25). NK-cells upregulate NKRs and maturate during the contact with self-MHC class I molecules (26). Contrary to this, NKR^+^CD8 T-cells express the least responsive Ly49 proteins, which is similar to murine TCR*β* ^+^CD8*αα* intestine intraepithelial lymphocytes and, to some extent, to NK T-cells [ (27, 28) and our unpublished observations].

The role of Ly49^+^CD8 T-cells in the immune system is uncertain. Several authors suggest that Ly49^+^CD8 T-cells have immunosuppressive properties (11, 12, 29, 30). At the same time, other studies suggest that CD8 T-cells upregulate Ly49 receptors as a response to TCR or cytokine stimulation under certain conditions, either because of exposure to autoantigens (10, 16) or during the antiviral response (13, 15, 31, 32). There is data claiming that NK1.1^+^CD8 T-cells include a significant fraction of Ly49^+^ cells and play an important role during the early stage of the immune response, show some innate-like features (33, 34) and impact the anticancer immunity significantly (35).

CD8 T-cells upregulate Ly49 receptors quite early during the response as 5 days after infection Ly49^+^CD8 T-cells account for approximately 10% of total IFN*γ*-producing CD8 T-cells (data not shown). Although the Ly49^+^CD8 T-cell pool is incomparably smaller than of the conventional CD8 T-cells and contracts faster, the antigen-specific Ly49^+^CD8 T-cells are detectable even 24 days post-infection. We also observed the broad antigen specificity of Ly49^+^CD8 T-cells generated during the infection (**Figures 4E** and **S2B**), which agrees with the polyclonal nature of Ly49^+^CD8 T-cells (8, 16). We did not perform more extensive experiments to assess the effector functions of Ly49^+^CD8 T-cells due to the limited number of this cell population even during the infection. However, some reports demonstrate cytotoxicity against the virus-infected target cells (13, 15).

To test whether Ly49^+^CD8 T-cells support or repress the antiviral response, we isolated and expanded Ly49^+^ and Ly49^−^ memory phenotype CD8 T-cells from LCMV-immune mice, expanded them *in vitro*, and transferred them into the naïve syngeneic hosts. Although we were not able to distinguish the transferred cells from the host’s cells, we can still judge the response and the virus load in tissues. Transferred Ly49^+^CD8 T-cells not only did not suppress the response, but the mice that received them had an increased amount of effector CD8 T-cells, decreased LCMV load in the spleen, and an increased number of IFN*γ* ^+^CD8 T-cells upon the peptide re-stimulation (**Figures 6A, D, F**).

Whether the expression of MHC class I specific NKRs gives additional survival benefits to CD8 T-cells is a matter of debate. KIR receptors expressed on human CD8 T-cells protect the host cells from activation-induced cell death under certain conditions (4, 6, 7, 36, 37). Transgenic expression of human KIR2DL3 receptor and the corresponding MHC class I ligand HLA-Cw3 in mice facilitates the accumulation of memory phenotype CD122^+^CD8 T-cells (38). Transgenic expression of Ly49 receptors on T-cell lymphoma cell lines protects them from the activation-induced cell death (39). However, CD8 T-cell-restricted Ly49 expression in mice shows conflicting results regarding the protection against activation-induced cell death (40).

We found no major differences in the phenotype peripheral T-cells in Ly49 KO mice (**Figure 5A**) and observed just a slightly higher proliferation of naïve CD8 T-cells from Ly49 KO mice during the polyclonal stimulation *in vitro* (**Figure 5B**). There were some defects in the primary antiviral response (**Figures 5D, F**, and **S3A**), but overall the response was impaired not critically. The antiviral response upon secondary re-encounter was also unimpaired (**Figure 6**), which suggests that the formation of the memory pool is not impaired because of the absence of Ly49 receptors. Thus, we conclude Ly49 receptors do not grant additional major benefits to CD8 T-cells. Although, we must be careful with these results as the lack of the whole cluster of Ly49 genes might affect all the cell types, which express them (NK-cells and NKT-cells), and more experiments are required (41).

The major factors affecting the upregulation of Ly49 receptors by CD8 T-cells include the immune response and STAT1 signaling (10, 13, 15, 16, 31). Immunization with MHC class II-restricted antigens also facilitates the upregulation of Ly49 receptors on CD8 T-cells, which suggests interactions with some other cells, presumably with activated T-cells (11, 12, 30). Further, some studies propose the involvement of CD4 T-cells as well as CD4 T-regs in the induction or survival of Ly49^+^CD8 T-cell (10, 42). Attenuation of IL-15 signaling in either IL-15 KO mice or mice deficient in IL-15 signaling components reduces the amount of Ly49^+^CD8 T-cells in mice (18, 19). Ly49^+^CD8 T-cells accumulate during the culture of enriched CD8 T-cells with IL-2 *in vitro* (43).

The lymphopenic environment itself cannot induce Ly49 receptors on CD8 T-cells, which is comparable to the previous studies (10, 15, 44). We also did not observe the dramatic accumulation of Ly49^+^CD8 T-cells in mice with age described by other researchers (2, 10), which might result from the differences in the animal housing conditions. These data also suggest that CD8 T-cells acquire Ly49 receptors post-thymically, and the most active accumulation of Ly49^+^CD8 T-cells takes place during sexual maturation.

We found valuable clues about the regulation of Ly49 receptors in mice from the *in vitro* stimulation and infection studies. CD8 T-cells stimulated *in vitro* demonstrate dynamic regulation and reversible expression of Ly49 receptors. CD8 T-cells stimulated *in vitro* demonstrate dynamic regulation and reversible expression of Ly49 receptors. Ly49^+^CD8 T-cells downregulate inhibitory Ly49 receptors (especially, Ly49F) and upregulate activating Ly49D+H receptors upon TCR engagement *in vitro* (**Figures 7A** and **S4A**). Stimulation with cytokines (IL-2 and/or IL-15) itself or combined with anti-CD3ϵ promotes the expression of inhibitory Ly49 receptors (IL-2 promotes the expression of Ly49G and Ly49A together with Ly49F, while IL-15 mostly promotes the expression of Ly49F), but only on a fraction of CD8 T-cells, which already expressed Ly49 receptors before. We were not able to induce inhibitory Ly49 receptors on conventional CD8 T-cells under the tested *in vitro* condition, but we observed a measurable upregulation of activating Ly49 receptors during TCR stimulation what generally is very similar to the effects observed with Ly49^+^CD8 T-cells (**Figures 7B, C**, **S4B, C**). Importantly, the results from LCMV infection experiments support our stimulation *in vitro* results. A fraction of the virus-specific CD8 T-cells express both inhibitory and activating Ly49 receptors and the major fraction of these Ly49^+^CD8 T-cells expresses inhibitory Ly49 receptors (**Figure 8C**).

The possible regulatory mechanism of NKR expression on CD8 T-cells is still elusive. In humans, inhibitors of DNA methylation added to CD8 T-cells during the TCR stimulation could promote KIR upregulation (8). Another study demonstrated the presence of distinct transcription machinery in human NK-cells and CD8 T-cells, which supports the expression of different spectrums of KIR receptors (45). Our data suggest a stepwise acquisition of Ly49 receptors upon CD8 T-cell activation. According to this, TCR stimulation induces activating Ly49 receptors. Common *γ*-chain cytokines (IL-2 and/or IL-15) and interactions with microenvironment affect the Ly49 expression supporting cells bearing Ly49 receptors and promoting the acquisition of inhibitory Ly49 receptors. This process depends on CD8 T-cells itself, while also requires factors delivered from the microenvironment of CD8 T-cells as well as IL-2 and/or IL-15. Some similarities with human KIR^+^CD8 T-cells may be noticed (46). Later, Ly49^+^CD8 T-cells can either downregulate Ly49 receptors during the following TCR stimulation, or the antigen-specific Ly49^+^CD8 T-cells may just vanish over time. These results broaden our understanding of the mechanism regulating MHC class I NK-cell receptor expression on CD8 T-cells and may be used to additionally unveil this process.

## Data Availability Statement

The raw data supporting the conclusions of this article will be made available by the authors, without undue reservation.

## Ethics Statement

The animal study was reviewed and approved by Review Committee of Zhejiang University School of Medicine.

## Author Contributions

DS, DR, and LL designed the research. DS, DR, DL, PW, MJ, MZ, and QX performed the experiments. DS, DR, and LL analyzed the data. DS, DR, PW, and LL wrote the paper. All authors contributed to the article and approved the submitted version.

## Funding

This work was supported by grants from the National Key R&D Program of China (2018YFC1105102), the National Natural Science Foundation of China (31770954, 31930038, 31530019 to LL, 81901605 to MZ, 31700766 to DS, and 31900628 to QX) and the Fundamental Research Funds for the Central Universities (2018XZZX001-12 to LL).

## Conflict of Interest

The authors declare that the research was conducted in the absence of any commercial or financial relationships that could be construed as a potential conflict of interest.

## Supplementary Material

Supplementary Figure 1 Gating strategy used to assess the repertoire of Ly49 receptors expressed on CD8 T-cells. Dead cells were excluded using staining with FVS dye and cell morphology. Splenocytes were stained with a mixture of antibodies against TCR*β*, CD8*α*, and Ly49 receptors **(A**, **D**, **H**, **G**, **F**, and **C/I)** and gated on CD8 T-cells (TCR*β* ^+^CD8*α* ^+^). Then the cells were divided stepwise according to expression of Ly49F or Ly49C/I, Ly49G, Ly49A, and Ly49D+H. Each population frequency was determined and calculated. Plots are representative of four mice.

Supplementary Figure 2 Ly49^+^CD8 T-cells produce IFN*γ* in response to the diverse array of viral peptides. **(A)** The percentage of Ly49^−^ memory phenotype CD8 T-cells and Ly49^+^CD8 T-cells among the total splenic CD8 T-cells at different time points after infection. The data is summarized from two independent experiments; each time point of each experiment has at least four mice. *P_(t)_ < 0.05 comparing to Ly49^+^CD8 T-cells. ^#^P_(t)_ < 0.05 comparing to the same type of CD8 T-cells at the previous time point. ^@^P_(t)_ < 0.05 comparing to the same type of CD8 T-cells from non-infected mice. **(B)** splenocytes were stimulated with the indicated virus peptides *in vitro* and stained for IFN*γ*. Representative pictures of IFN*γ* expression among each type of CD8 T-cells at different time points are shown. Each time point of includes at least four mice.

Supplementary Figure 3 CD8 T-cells from Ly49 KO show reduced upregulation of KLRG1 during LCMV infection, but do not have major defects in cytokine production in response to major immunodominant viral peptides. **(A)** Surface phenotype and the relative amount KLRG1^+^ cells among the splenic memory phenotype CD8 T-cells from intact or LCMV-infected (day 8) WT or Ly49 KO mice (day 8). Representative plots and the relative amount of KLRG1^+^ cells among the memory phenotype CD8 T-cells of LCMV-infected mice (day 8) are shown. The data are summarized from two experiments with each group containing at least four mice. *P(t) < 0.05 comparing to WT mice. **(B)** *IFNγ* and TNFα expression by splenic CD8 T-cells from LCMV-infected (day 8) WT or Ly49 KO mice in response to the indicated peptides. Representative pictures and the relative amount of IFN*γ* ^+^ (**C**) or TNFα+ **(D)**. ^#^P(t) < 0.05 comparing to the same type of CD8 T-cells stimulated with gp33. The data is representative of two experiments; each experimental group has at least four mice.

Supplementary Figure 4 Expression of Ly49 receptors on FACS-purified Ly49^+^ **(A)**, naïve **(B)**, and memory phenotype **(C)** CD8 T-cells stimulated at the indicated conditions *in vitro.* Representative pictures of one out of six experiments are shown.

## Abbreviations

nCD8, naïve CD8 T-cells; MP CD8, memory phenotype CD8 T-cells; tCD8, total CD8 T-cells; nCD4, naïve CD4 T-cells; CD4 T-regs, regulatory CD4 T-cells; tCD4, total CD4 T-cells; day p.i., day post infection; LCMV, lymphocytic choriomeningitis virus.
