# Supplementary material for: Functional Characterization of Ly49+CD8 T-Cells in Both Normal Condition and During Anti-Viral Response
Source: Front Immunol. 2021 Jan 7;11:602783. doi: 10.3389/fimmu.2020.602783 (PMC7817614; doi:10.3389/fimmu.2020.602783)
Supplement: Supplementary Figure 1 — Gating strategy used to assess the repertoire of Ly49 receptors expressed on CD8 T-cells. Dead cells were excluded using staining with FVS dye and cell morphology. Splenocytes were stained with a mixture of antibodies against TCRβ, CD8α, and Ly49 receptors (A, D, H, G, F, and C/I) and gated on CD8 T-cells (TCRβ +CD8α +). Then the cells were divided stepwise according to expression of Ly49F or Ly49C/I, Ly49G, Ly49A, and Ly49D+H. Each population frequency was determined and calculated. Plots are representative of four mice. [file Presentation_1.pptx]

## Slide 1
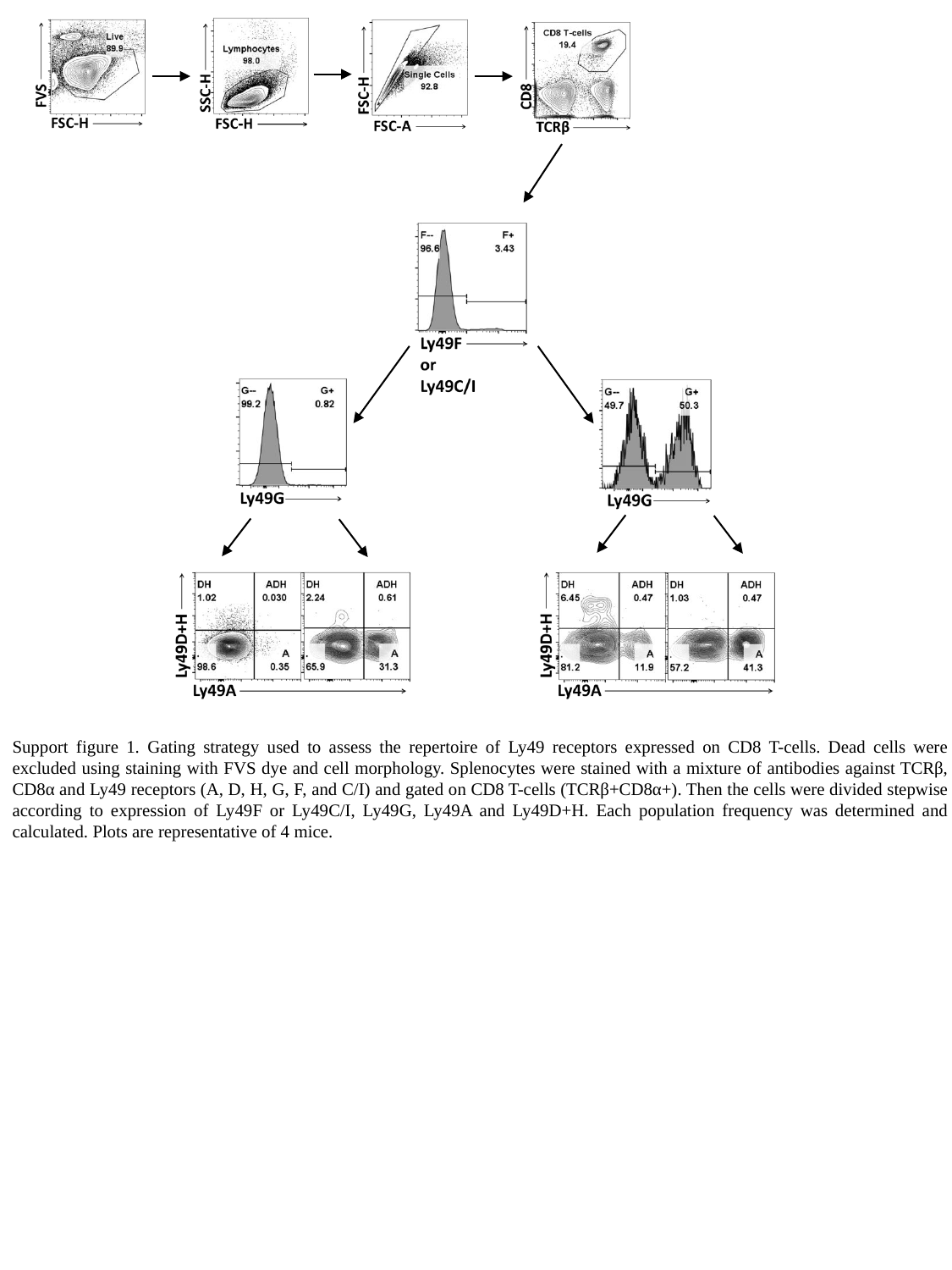

Support figure 1. Gating strategy used to assess the repertoire of Ly49 receptors expressed on CD8 T-cells. Dead cells were excluded using staining with FVS dye and cell morphology. Splenocytes were stained with a mixture of antibodies against TCRβ, CD8α and Ly49 receptors (A, D, H, G, F, and C/I) and gated on CD8 T-cells (TCRβ+CD8α+). Then the cells were divided stepwise according to expression of Ly49F or Ly49C/I, Ly49G, Ly49A and Ly49D+H. Each population frequency was determined and calculated. Plots are representative of 4 mice.

## Slide 2
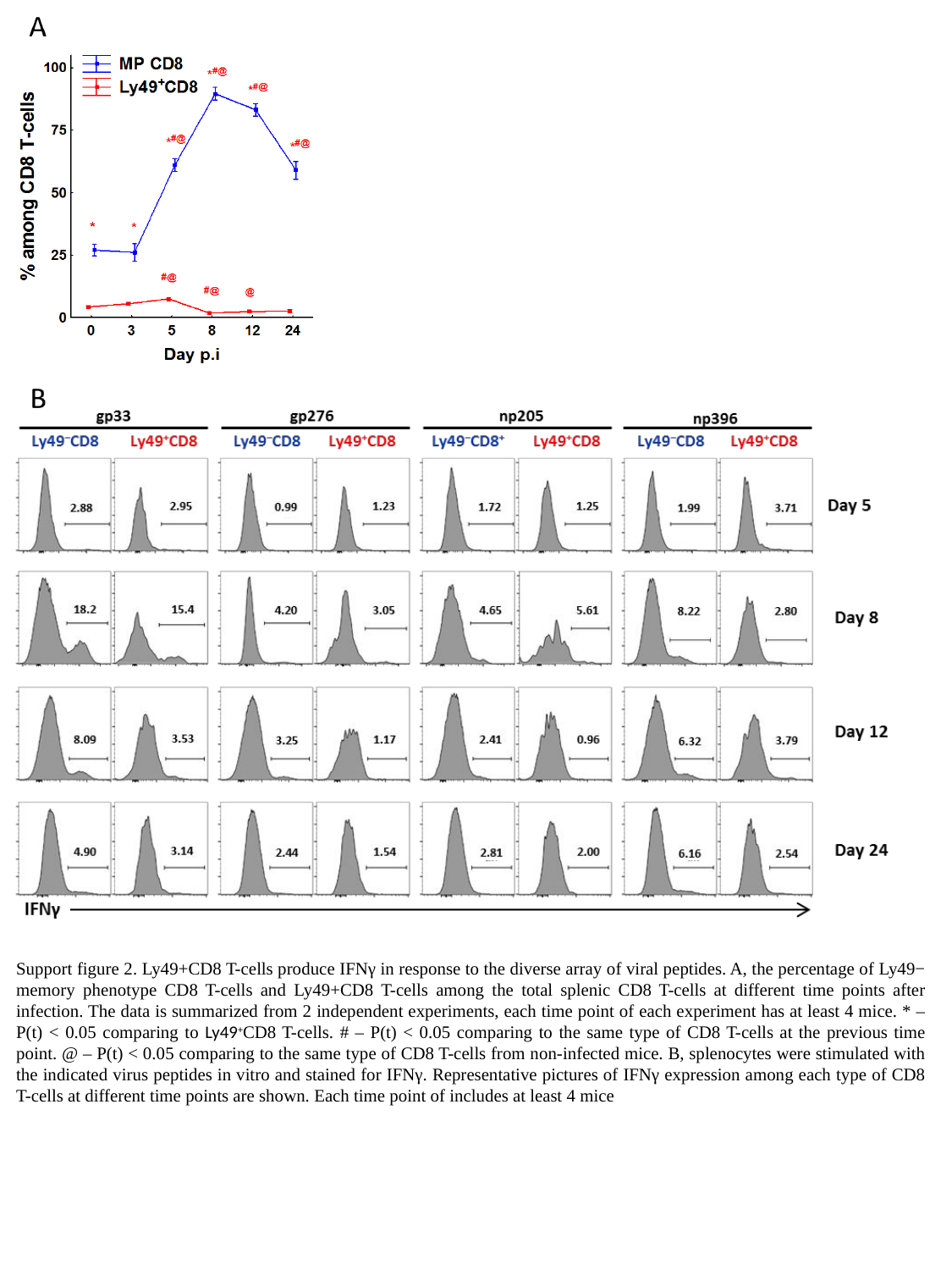

Support figure 2. Ly49+CD8 T-cells produce IFNγ in response to the diverse array of viral peptides. A, the percentage of Ly49− memory phenotype CD8 T-cells and Ly49+CD8 T-cells among the total splenic CD8 T-cells at different time points after infection. The data is summarized from 2 independent experiments, each time point of each experiment has at least 4 mice. * – P(t) < 0.05 comparing to Ly49+CD8 T-cells. # – P(t) < 0.05 comparing to the same type of CD8 T-cells at the previous time point. @ – P(t) < 0.05 comparing to the same type of CD8 T-cells from non-infected mice. B, splenocytes were stimulated with the indicated virus peptides in vitro and stained for IFNγ. Representative pictures of IFNγ expression among each type of CD8 T-cells at different time points are shown. Each time point of includes at least 4 mice

## Slide 3
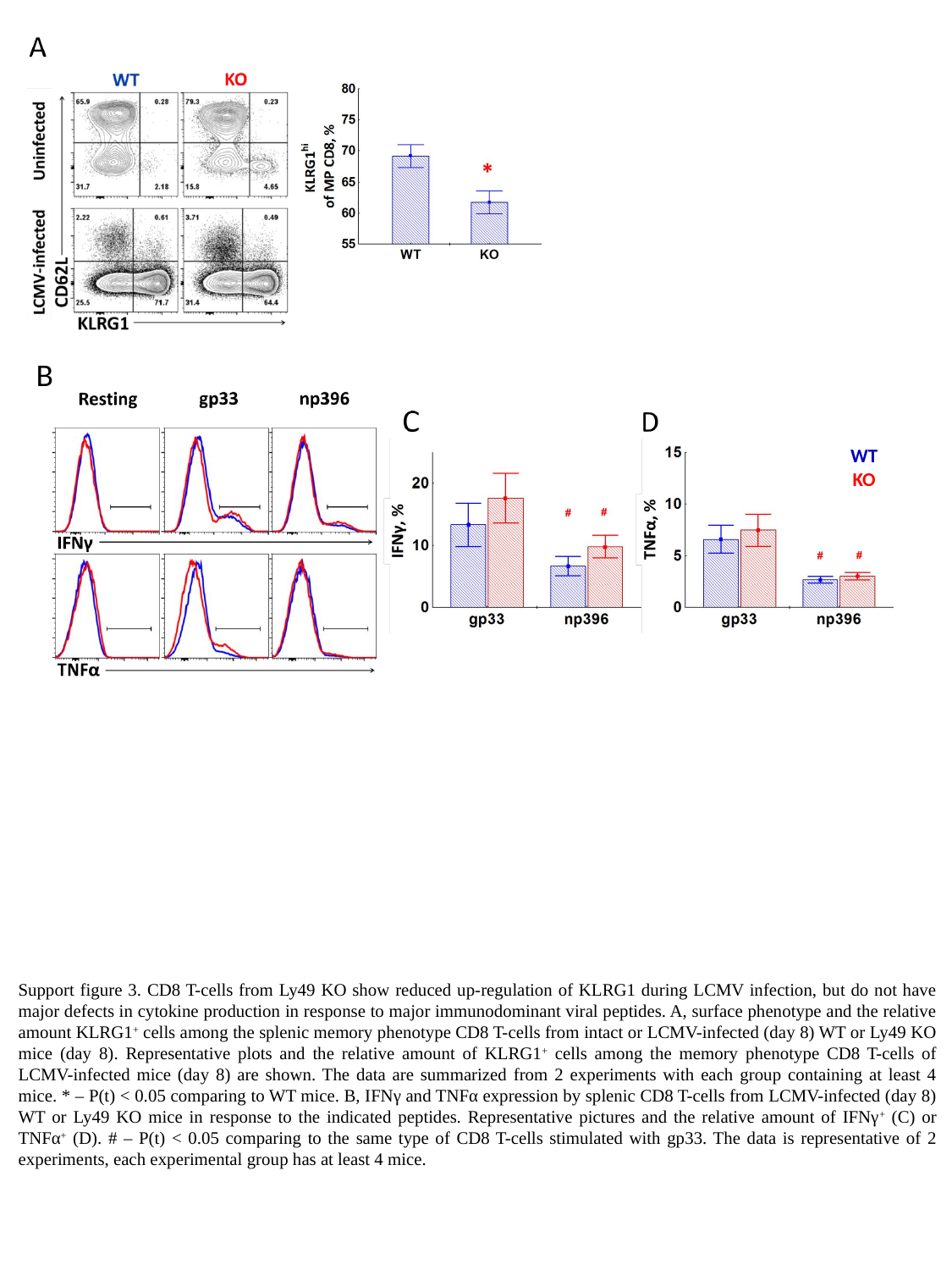

B
WT
KO
Support figure 3. CD8 T-cells from Ly49 KO show reduced up-regulation of KLRG1 during LCMV infection, but do not have major defects in cytokine production in response to major immunodominant viral peptides. A, surface phenotype and the relative amount KLRG1+ cells among the splenic memory phenotype CD8 T-cells from intact or LCMV-infected (day 8) WT or Ly49 KO mice (day 8). Representative plots and the relative amount of KLRG1+ cells among the memory phenotype CD8 T-cells of LCMV-infected mice (day 8) are shown. The data are summarized from 2 experiments with each group containing at least 4 mice. * – P(t) < 0.05 comparing to WT mice. B, IFNγ and TNFα expression by splenic CD8 T-cells from LCMV-infected (day 8) WT or Ly49 KO mice in response to the indicated peptides. Representative pictures and the relative amount of IFNγ+ (C) or TNFα+ (D). # – P(t) < 0.05 comparing to the same type of CD8 T-cells stimulated with gp33. The data is representative of 2 experiments, each experimental group has at least 4 mice.

## Slide 4
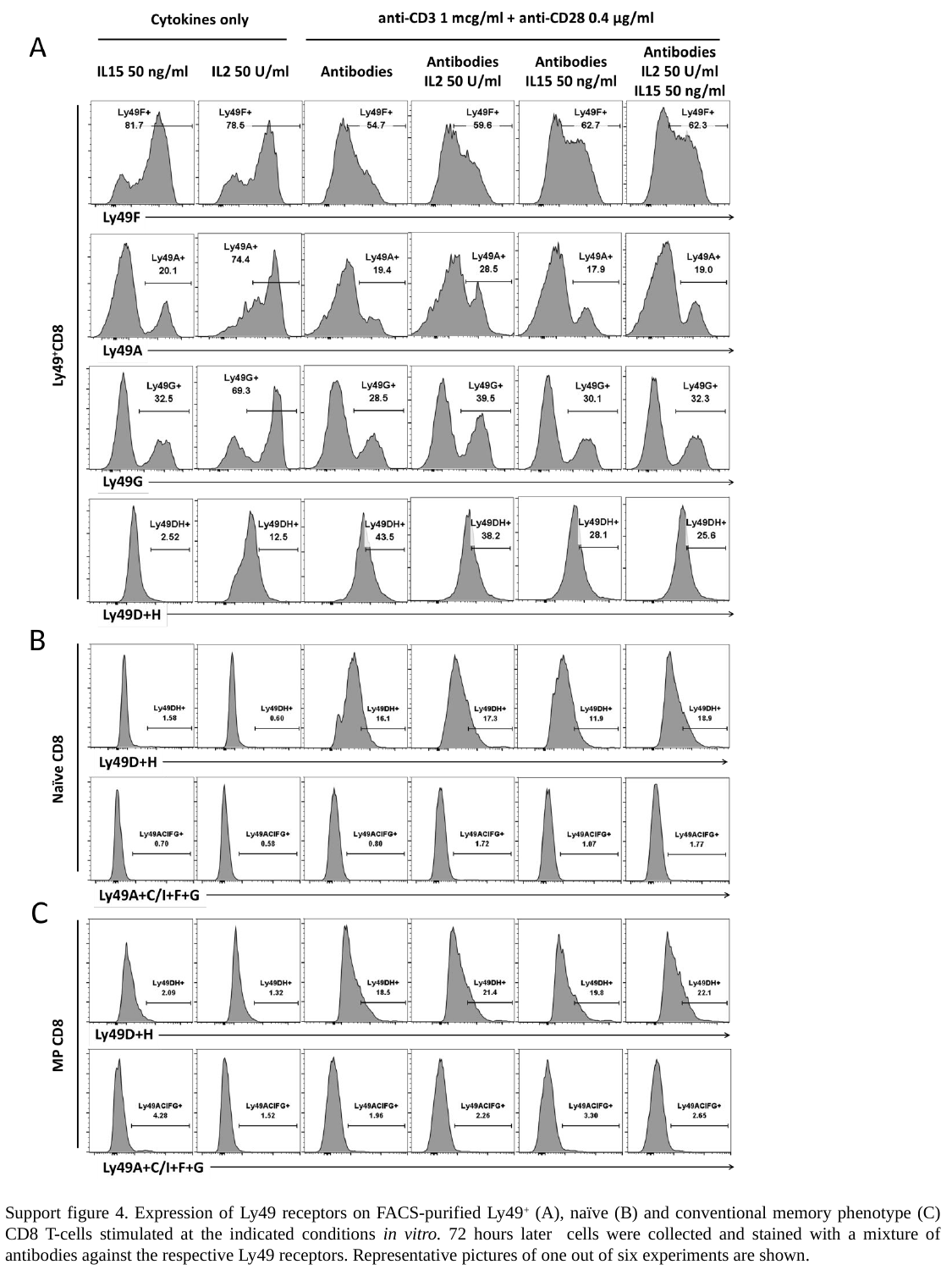

B
Support figure 4. Expression of Ly49 receptors on FACS-purified Ly49+ (A), naïve (B) and conventional memory phenotype (C) CD8 T-cells stimulated at the indicated conditions in vitro. 72 hours later cells were collected and stained with a mixture of antibodies against the respective Ly49 receptors. Representative pictures of one out of six experiments are shown.

## Slide 5
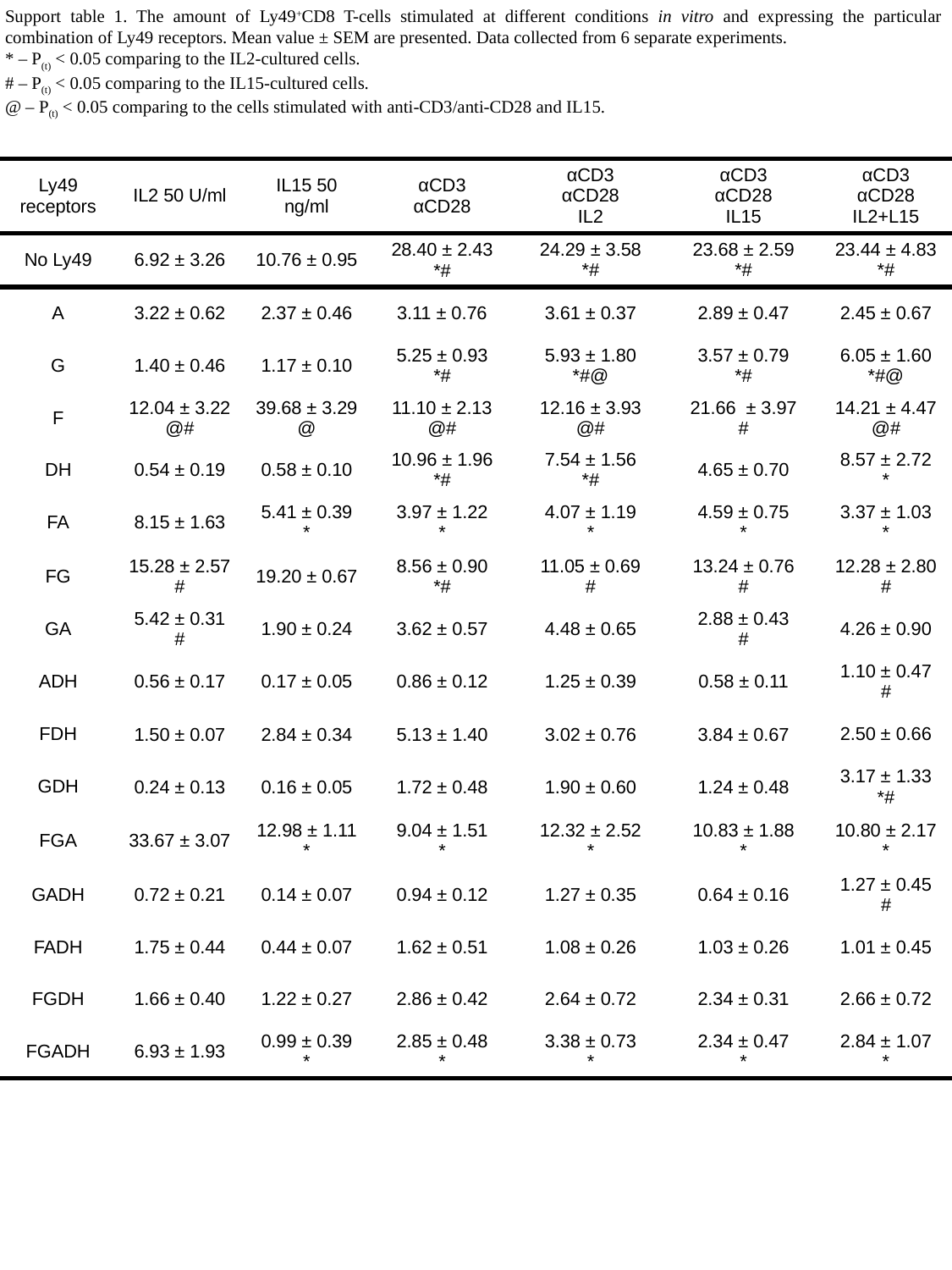

Support table 1. The amount of Ly49+CD8 T-cells stimulated at different conditions in vitro and expressing the particular combination of Ly49 receptors. Mean value ± SEM are presented. Data collected from 6 separate experiments.
* – P(t) < 0.05 comparing to the IL2-cultured cells.
# – P(t) < 0.05 comparing to the IL15-cultured cells.
@ – P(t) < 0.05 comparing to the cells stimulated with anti-CD3/anti-CD28 and IL15.
| Ly49 receptors | IL2 50 U/ml | IL15 50 ng/ml | αCD3 αCD28 | αCD3 αCD28 IL2 | αCD3 αCD28 IL15 | αCD3 αCD28 IL2+L15 |
| --- | --- | --- | --- | --- | --- | --- |
| No Ly49 | 6.92 ± 3.26 | 10.76 ± 0.95 | 28.40 ± 2.43 \*# | 24.29 ± 3.58 \*# | 23.68 ± 2.59 \*# | 23.44 ± 4.83 \*# |
| A | 3.22 ± 0.62 | 2.37 ± 0.46 | 3.11 ± 0.76 | 3.61 ± 0.37 | 2.89 ± 0.47 | 2.45 ± 0.67 |
| G | 1.40 ± 0.46 | 1.17 ± 0.10 | 5.25 ± 0.93 \*# | 5.93 ± 1.80 \*#@ | 3.57 ± 0.79 \*# | 6.05 ± 1.60 \*#@ |
| F | 12.04 ± 3.22 @# | 39.68 ± 3.29 @ | 11.10 ± 2.13 @# | 12.16 ± 3.93 @# | 21.66 ± 3.97 # | 14.21 ± 4.47 @# |
| DH | 0.54 ± 0.19 | 0.58 ± 0.10 | 10.96 ± 1.96 \*# | 7.54 ± 1.56 \*# | 4.65 ± 0.70 | 8.57 ± 2.72 \* |
| FA | 8.15 ± 1.63 | 5.41 ± 0.39 \* | 3.97 ± 1.22 \* | 4.07 ± 1.19 \* | 4.59 ± 0.75 \* | 3.37 ± 1.03 \* |
| FG | 15.28 ± 2.57 # | 19.20 ± 0.67 | 8.56 ± 0.90 \*# | 11.05 ± 0.69 # | 13.24 ± 0.76 # | 12.28 ± 2.80 # |
| GA | 5.42 ± 0.31 # | 1.90 ± 0.24 | 3.62 ± 0.57 | 4.48 ± 0.65 | 2.88 ± 0.43 # | 4.26 ± 0.90 |
| ADH | 0.56 ± 0.17 | 0.17 ± 0.05 | 0.86 ± 0.12 | 1.25 ± 0.39 | 0.58 ± 0.11 | 1.10 ± 0.47 # |
| FDH | 1.50 ± 0.07 | 2.84 ± 0.34 | 5.13 ± 1.40 | 3.02 ± 0.76 | 3.84 ± 0.67 | 2.50 ± 0.66 |
| GDH | 0.24 ± 0.13 | 0.16 ± 0.05 | 1.72 ± 0.48 | 1.90 ± 0.60 | 1.24 ± 0.48 | 3.17 ± 1.33 \*# |
| FGA | 33.67 ± 3.07 | 12.98 ± 1.11 \* | 9.04 ± 1.51 \* | 12.32 ± 2.52 \* | 10.83 ± 1.88 \* | 10.80 ± 2.17 \* |
| GADH | 0.72 ± 0.21 | 0.14 ± 0.07 | 0.94 ± 0.12 | 1.27 ± 0.35 | 0.64 ± 0.16 | 1.27 ± 0.45 # |
| FADH | 1.75 ± 0.44 | 0.44 ± 0.07 | 1.62 ± 0.51 | 1.08 ± 0.26 | 1.03 ± 0.26 | 1.01 ± 0.45 |
| FGDH | 1.66 ± 0.40 | 1.22 ± 0.27 | 2.86 ± 0.42 | 2.64 ± 0.72 | 2.34 ± 0.31 | 2.66 ± 0.72 |
| FGADH | 6.93 ± 1.93 | 0.99 ± 0.39 \* | 2.85 ± 0.48 \* | 3.38 ± 0.73 \* | 2.34 ± 0.47 \* | 2.84 ± 1.07 \* |
